# Supplementary material for: Structural insights into the mechanism of overcoming Erm-mediated resistance by macrolides acting together with hygromycin-A
Source: Nat Commun. 2023 Jul 14;14:4196. doi: 10.1038/s41467-023-39653-5 (PMC10349075; doi:10.1038/s41467-023-39653-5)
Supplement: Supplementary file 1 — Supplementary Information [file 41467_2023_39653_MOESM1_ESM.pdf]

I. SUPPLEMENTARY FIGURES

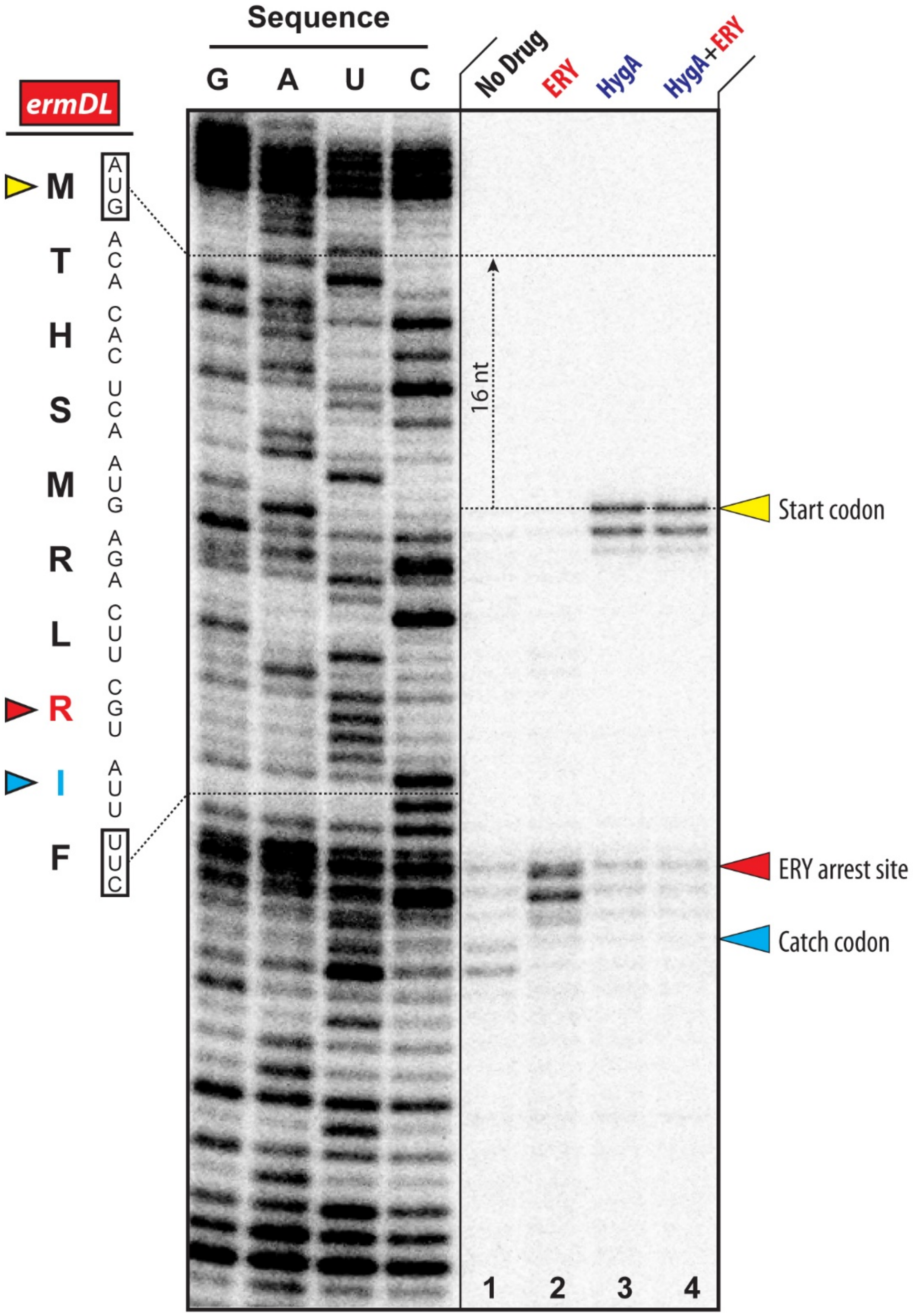

Supplementary Figure 1 | Inhibitory effect of the HygA-ERY pair on mRNA translation. Ribosome stalling caused by HygA (100 µM, lane 2), ERY (50 µM, lane 3), or their combination (lane 4) on *ermDL* mRNA template revealed by reverse transcription inhibition (toe-printing) assay in a recombinant cell-free translation system. Nucleotide sequence of *ermDL* gene, and the corresponding amino acid sequence, are shown on the left. Sequencing lanes (G, A, U, C) are on the left of the gel. Due to the large size of the

ribosome, the reverse transcriptase used in the toe-printing assay stops 16 nucleotides downstream of the codon located in the P site. Yellow arrowhead marks the translation start site, which is also the site of HygA-induced arrest. ERY-induced arrest site is marked with red arrowhead. The toe-printing reactions were also supplemented with mupirocin, an inhibitor of isoleucyl-tRNA-synthetase, to arrest all translating ribosomes that bypassed drug-specific stall sites at the downstream isoleucyl codon (blue arrowhead). The toe-printing assay was repeated twice with similar results. Source data are provided as a Source Data file. Note that, similar to HygA alone, HygA-ERY pair stalls ribosome at the translation start site (yellow arrowhead).

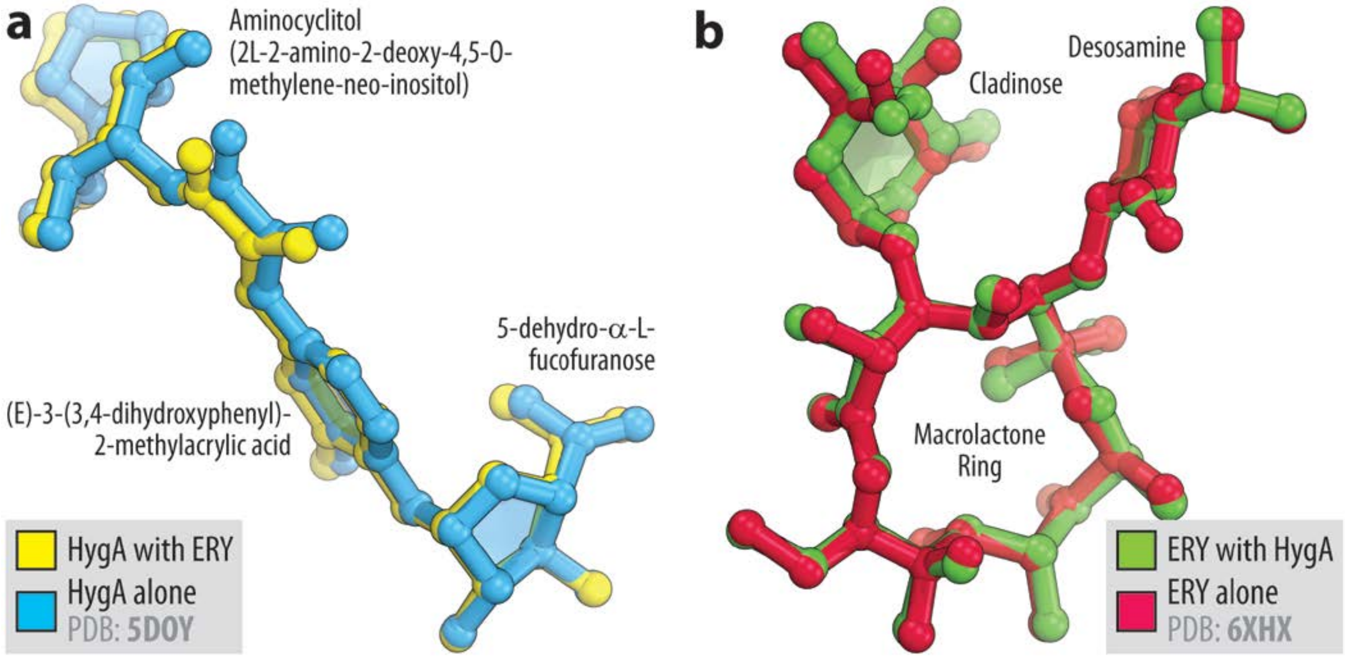

Supplementary Figure 2 | Comparison of the binding sites of hygromycin A and erythromycin bound to the ribosome individually and in combination. (**a, b**) Superposition of the new structure of HygA-ERY pair (yellow and green) with the previous structures of ribosome-bound individual HygA (a, blue, PDB entry 5DOY[1] [https://doi.org/10.2210/pdb5DOY/pdb]) or ERY (b, crimson, PDB entry 6XHX[2] [https://doi.org/10.2210/pdb6XHX/pdb]). All structures were aligned based on domain V of the 23S rRNA. Note that the binding sites of HygA and ERY are nearly identical regardless of whether the drugs are bound to the 70S ribosome alone or in combination with each other.

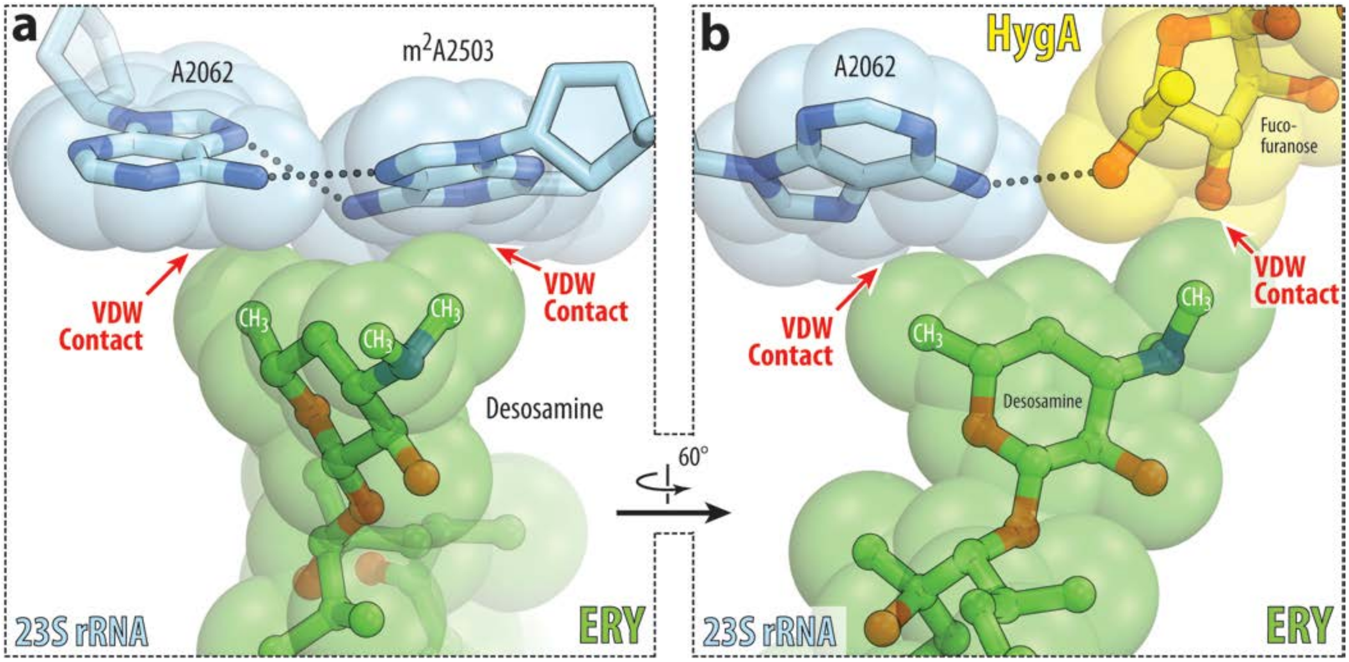

Supplementary Figure 3 | Van der Waals contacts of erythromycin with the A2062-A2503 Hoogsteen base-pair and hygromycin A. (**a**) VDW contacts of the methyl groups of ERY's desosamine (green) with the aromatic nucleobases A2062 and m^{2}A2503 of the 23S rRNA (light blue), which are involved in Hoogsteen base-pair formation. H-bond interactions are indicated with black dotted lines. (**b**) VDW interaction of dimethylamino group of desosamine of ERY with the fucofuranose moiety of HygA (yellow).

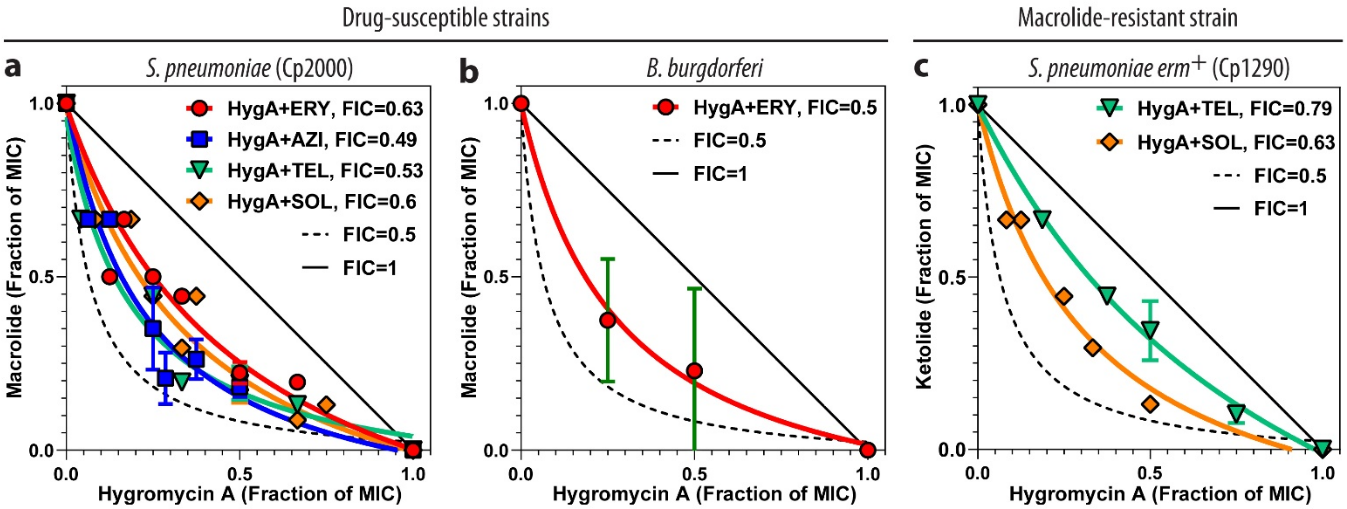

Supplementary Figure 4 | Antimicrobial effects of hygromycin A in combination with macrolides against susceptible and resistant bacteria. (**a-c**) Graphical representations of the results of the checkerboard assay using non-linear regression fit of the experimental data obtained for combinations of hygromycin A (HygA) with macrolides against *S. pneumoniae* WT strain Cp2000 (a), *B. burgdorferi* WT strain (b), or macrolide-resistant *S. pneumoniae* strain Cp1290 constitutively expressing Erm-methyltransferase (c). Graphs for combinations of HygA with macrolides erythromycin (ERY) or azithromycin (AZI) are shown in red and blue, respectively. Graphs for ketolides telithromycin (TEL) or solithromycin (SOL) are teal and orange, respectively. Solid black line represent the FIC index value of 1; dashed black curve – FIC index value of 0.5. The minimal observed FIC index values for each combination are shown in the legends. The corresponding MIC values are shown in **Supplementary Table 2**. Error bars show the mean standard deviation of 2-6 independent measurements. Source data are provided as a Source Data file. Note that most of the combinations of HygA with macrolides exhibit strong antimicrobial cooperativity (FIC≤1, below solid black line), which is nevertheless above the threshold for synergy (FIC≤0.5, above dashed black curve).

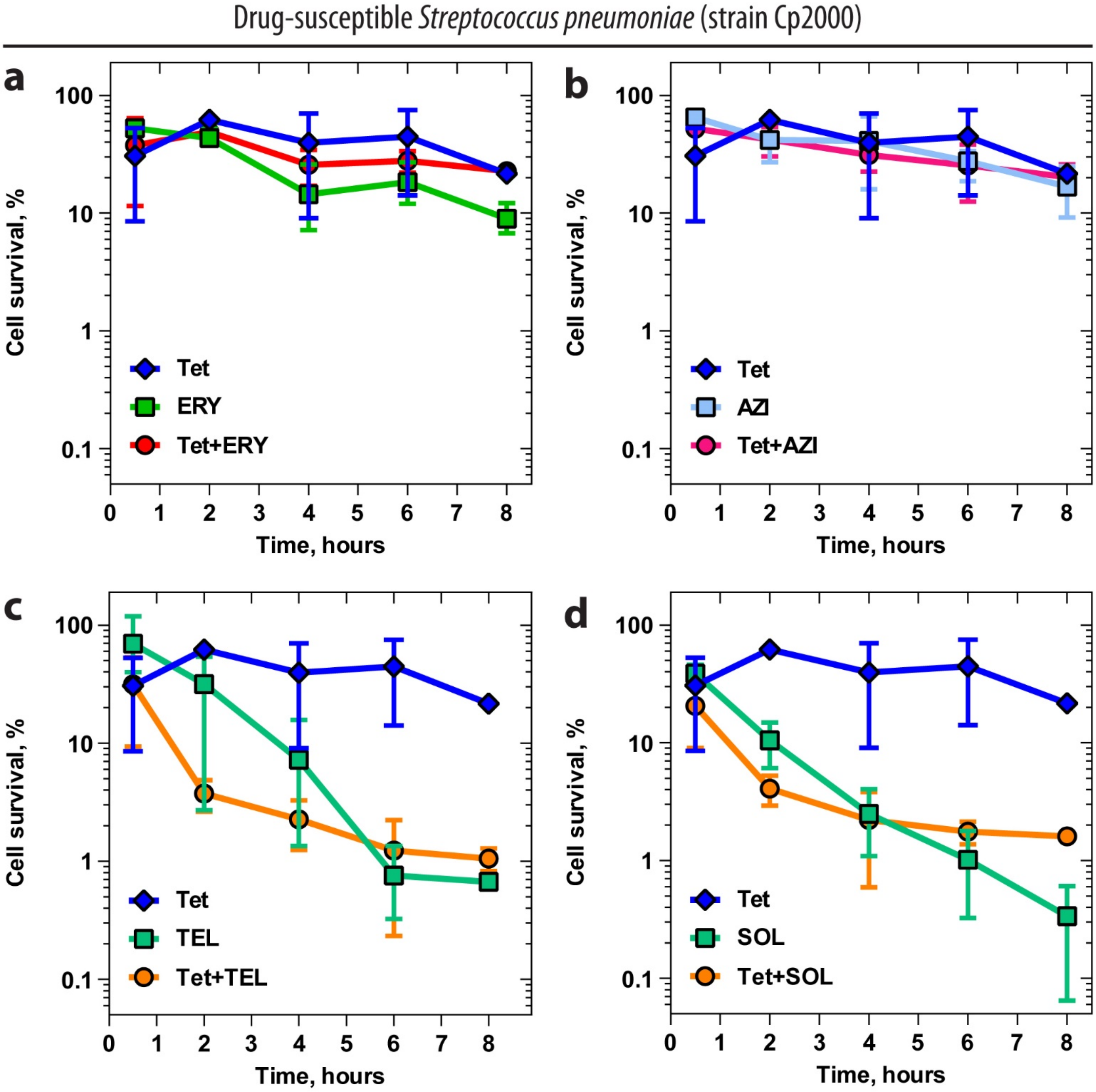

Supplementary Figure 5 | Tetracycline does not enhance killing activity of macrolides and ketolides. (**a-d**) Time-kill assays using drug-susceptible Cp2000 strain of *S. pneumoniae* exposed during various times to antibiotic concentrations at 4x MIC of tetracycline (Tet, blue plot, 0.6 µg/ml), macrolides erythromycin (a, ERY, green plot, 0.25 µg/ml), azithromycin (b, AZI, light blue plot, 0.5 µg/ml), or ketolides telithromycin (c, TEL, teal plot, 0.04 µg/ml) or solithromycin (d, SOL, teal plot, 0.04 µg/ml) alone and in combination with Tet (red, magenta, and orange plots, respectively). The initial number of viable cells (colony-forming units, CFUs) before the addition of drugs was arbitrarily assigned to 100%.

Error bars show the mean standard deviation of 2-5 independent measurements. Source data are provided as a Source Data file.

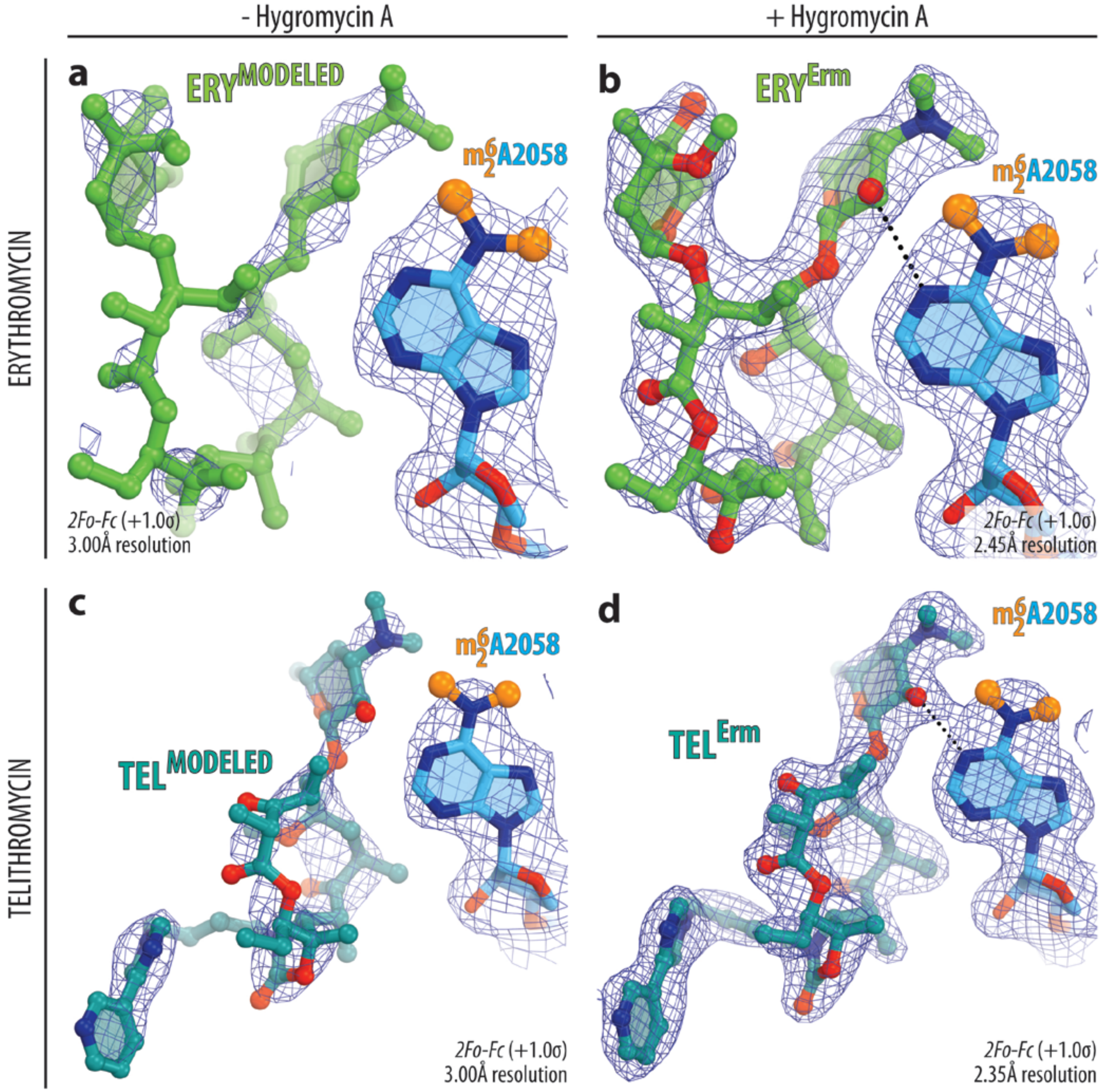

Supplementary Figure 6 | Electron density maps of macrolides bound to the Erm-modified *T. thermophilus* 70S ribosome. (**a-d**) $2F_o$-F_c electron difference Fourier maps of macrolide erythromycin (a, b, ERY, green) or ketolide telithromycin (c, d, TEL, teal) bound to the Erm-methylated 70S ribosome containing m^6_2A2058 residue (blue with methyl groups highlighted in orange) in the absence (a, c) or presence (b, d) of hygromycin A. The refined models of antibiotics are displayed in their respective electron density maps after the refinement (blue mesh). The overall resolution of the corresponding

structures and the contour levels of the depicted electron density maps are shown at the bottom of each panel.

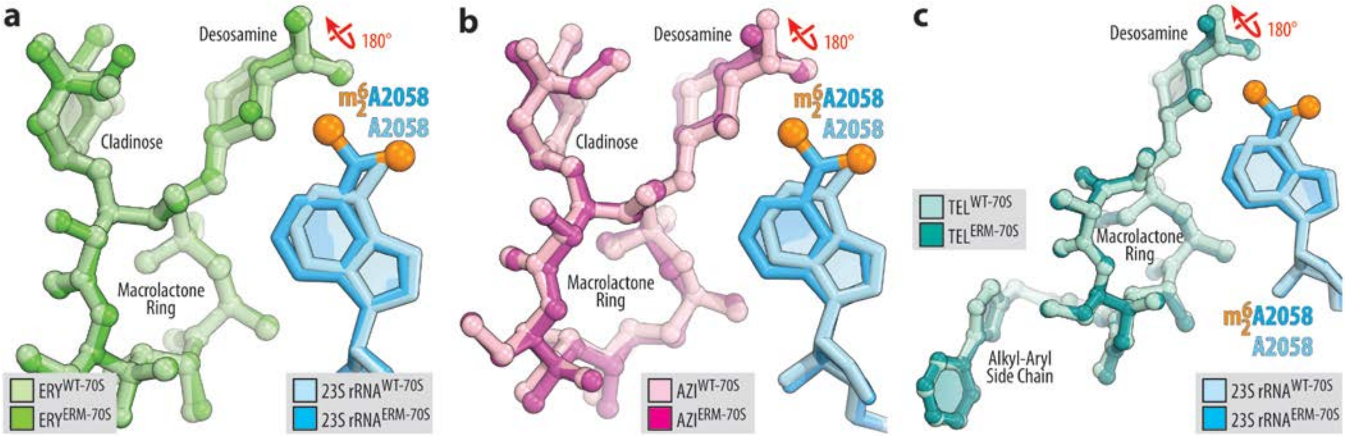

Supplementary Figure 7 | Comparisons of the structures of macrolides in the HygA-macrolide pairs bound to the Erm-modified *vs.* WT 70S ribosomes. (**a-c**) Superposition of the new structures of ERY (a), AZI (b), or TEL (c) in their complexes with WT 70S ribosome containing unmodified residue A2058 (light blue), with the new structures of the same drugs in their complexes with the Erm-modified 70S ribosome containing N6-dimethylated residue A2058 (blue with methyl groups highlighted in orange), all obtained in the presence of HygA (not shown). Note that while the overall positions of ribosome-bound macrolides are almost indistinguishable in each pair of structures, N6-dimethylation of A2058 results in a 180-degree rotation of the dimethylamino group of desosamine away from A2058 nucleobase towards the fucofuranose moiety of HygA and formation of a direct H-bond with it (red arrow).

II. SUPPLEMENTARY TABLES

Supplementary Table 1 | X-ray data collection and refinement statistics.

	WT A2058-unmethylated 70S-PY complex with HygA and ERY<br>PDB entry 8FC1	WT A2058-unmethylated 70S-PY complex with HygA and AZI<br>PDB entry 8FC2	WT A2058-unmethylated 70S-PY complex with HygA and TEL<br>PDB entry 8FC3
**Data collection**			
Space group	$P2_12_12_1$	$P2_12_12_1$	$P2_12_12_1$
Cell dimensions			
a, *b*, *c* (Å)	208.57, 448.58, 617.47	209.90, 450.73, 622.70	209.12, 449.23, 619.30
α, β, γ (°)	90.0, 90.0, 90.0	90.0, 90.0, 90.0	90.0, 90.0, 90.0
Resolution (Å)	224-2.50 (2.56-2.50)[a]	199-2.50 (2.56-2.50)[a]	188-2.60 (2.67-2.60)[a]
R_{merge}	16.9 (240.2)	18.5 (207.6)	16.7 (228.1)
$I / \sigma I$	9.13 (0.86)[b]	8.53 (1.00)[c]	7.36 (0.93)[d]
Completeness (%)	99.9 (100.0)	99.8 (99.7)	100.0 (100.0)
Redundancy	6.97 (7.21)	6.88 (6.99)	6.06 (6.17)
**Refinement**			
Resolution (Å)	2.50	2.50	2.60
No. reflections	1,965,409	2,001,911	1,761,497
R_{work} / R_{free}	20.9/24.6	20.7/25.0	20.1/24.5
No. atoms			
Protein	93,036	93,036	93,036
Ligand/ion	196,114	196,107	196,118
Water	7,823	7,575	7,947
B factors			
Protein	67.8	65.1	57.5
Ligand/ion	64.4	61.8	53.9
Water	49.5	46.1	38.7
R.m.s. deviations			
Bond lengths (Å)	0.004	0.004	0.004
Bond angles (°)	0.883	0.881	0.882

Values in parentheses are for the highest-resolution shell.
[a] Diffraction data from a **single crystal** were used to obtain the structure.
[b] I/σI = 2 at 2.75Å resolution.
[c] I/σI = 2 at 2.75Å resolution.
[d] I/σI = 2 at 2.85Å resolution.

Supplementary Table 1 | X-ray data collection and refinement statistics (continued).

	A2058-N6-dimethylated 70S-PY complex with HygA and ERY PDB entry 8FC4	A2058-N6-dimethylated 70S-PY complex with HygA and AZI PDB entry 8FC5	A2058-N6-dimethylated 70S-PY complex with HygA and TEL PDB entry 8FC6
**Data collection**			
Space group	$P2_12_12_1$	$P2_12_12_1$	$P2_12_12_1$
Cell dimensions			
a, *b*, *c* (Å)	209.46, 449.56, 619.15	209.55, 449.77, 619.46	209.60, 449.65, 619.88
α, β, γ (°)	90.0, 90.0, 90.0	90.0, 90.0, 90.0	90.0, 90.0, 90.0
Resolution (Å)	162-2.45 (2.51-2.45)[a]	225-2.65 (2.72-2.65)[a]	211-2.35 (2.41-2.35)[a]
R_{merge}	14.8 (156.9)	20.3 (214.8)	20.6 (277.1)
$I / \sigma I$	7.51 (0.85)[b]	8.10 (0.98)[c]	6.95 (0.82)[d]
Completeness (%)	98.8 (97.8)	99.8 (99.9)	99.8 (99.9)
Redundancy	4.21 (4.16)	6.84 (6.98)	6.92 (7.08)
**Refinement**			
Resolution (Å)	2.45	2.65	2.35
No. reflections	2,081,650	1,667,888	2,387,888
R_{work} / R_{free}	21.8/26.4	19.4/23.6	21.1/24.8
No. atoms			
Protein	93,036	93,036	93,036
Ligand/ion	195,936	196,078	196,121
Water	8,272	7,755	8,855
B factors			
Protein	64.4	66.4	60.8
Ligand/ion	61.1	62.0	57.4
Water	46.3	48.1	45.7
R.m.s. deviations			
Bond lengths (Å)	0.004	0.004	0.004
Bond angles (°)	0.892	0.873	0.905

Values in parentheses are for the highest-resolution shell.
[a] Diffraction data from a **single crystal** were used to obtain the structure.
[b] I/σI = 2 at 2.70Å resolution.
[c] I/σI = 2 at 2.85Å resolution.
[d] I/σI = 2 at 2.60Å resolution.

Supplementary Table 2 | Minimal inhibitory concentrations (MICs) of various antibiotics used in this study. Shown are MICs for hygromycin A and macrolides/ketolides (μg/ml) against drug-susceptible Cp2000 and *erm*-positive macrolide-resistant Cp1290 strains of *Streptococcus pneumoniae* as well as drug-susceptible strain B1286GFP of spirochete *Borrelia burgdorferi*. HygA – hygromycin A, ERY – erythromycin, AZI – azithromycin, TEL – telithromycin, SOL – solithromycin, TET – tetracycline, N/D – not determined.

Strain	MIC, μg/ml					
	HygA	ERY	AZI	TEL	SOL	TET
S. pneumoniae Cp2000	5	0.0625	0.125	0.01	0.01	0.15
S. pneumoniae Cp1290 (*erm*$^{+}$)	5	N/D	N/D	0.5	1.0	N/D
B. burgdorferi B1286GFP	0.25	0.004	N/D	N/D	N/D	N/D

III. SUPPLEMENTARY REFERENCES

1. Polikanov, Y.S. et al. Distinct tRNA accommodation intermediates observed on the ribosome with the antibiotics hygromycin A and A201A. *Mol. Cell* **58**, 832-844 (2015).

2. Svetlov, M.S. et al. Structure of Erm-modified 70S ribosome reveals the mechanism of macrolide resistance. *Nat. Chem. Biol.* **17**, 412-420 (2021).
