# Supplementary material for: Structural insights into the mechanism of overcoming Erm-mediated resistance by macrolides acting together with hygromycin-A
Source: Nat Commun. 2023 Jul 14;14:4196. doi: 10.1038/s41467-023-39653-5 (PMC10349075; doi:10.1038/s41467-023-39653-5)
Supplement: Supplementary file 3 — Reporting Summary [file 41467_2023_39653_MOESM3_ESM.pdf]

nature portfolio

Corresponding author(s): Yury Polikanov & Maxim Svetlov

Last updated by author(s): 05/31/2023

Reporting Summary

Nature Portfolio wishes to improve the reproducibility of the work that we publish. This form provides structure for consistency and transparency in reporting. For further information on Nature Portfolio policies, see our Editorial Policies and the Editorial Policy Checklist.

Statistics

For all statistical analyses, confirm that the following items are present in the figure legend, table legend, main text, or Methods section.

n/a	Confirmed	
☐	☒	The exact sample size (n) for each experimental group/condition, given as a discrete number and unit of measurement
☒	☐	A statement on whether measurements were taken from distinct samples or whether the same sample was measured repeatedly
☒	☐	The statistical test(s) used AND whether they are one- or two-sided<br>*Only common tests should be described solely by name; describe more complex techniques in the Methods section.*
☒	☐	A description of all covariates tested
☒	☐	A description of any assumptions or corrections, such as tests of normality and adjustment for multiple comparisons
☐	☒	A full description of the statistical parameters including central tendency (e.g. means) or other basic estimates (e.g. regression coefficient) AND variation (e.g. standard deviation) or associated estimates of uncertainty (e.g. confidence intervals)
☒	☐	For null hypothesis testing, the test statistic (e.g. F, t, r) with confidence intervals, effect sizes, degrees of freedom and P value noted<br>*Give P values as exact values whenever suitable.*
☒	☐	For Bayesian analysis, information on the choice of priors and Markov chain Monte Carlo settings
☒	☐	For hierarchical and complex designs, identification of the appropriate level for tests and full reporting of outcomes
☒	☐	Estimates of effect sizes (e.g. Cohen's d, Pearson's r), indicating how they were calculated

Our web collection on statistics for biologists contains articles on many of the points above.

Software and code

Policy information about availability of computer code

Data collection	X-ray diffraction data was collected at beamlines 24ID-C and 24ID-E at the Advanced Photon Source (Argonne National Laboratory) using NE-CAT Remote Access software v6.2.0.
Data analysis	Raw X-ray crystallographic data were integrated and scaled using XDS software (Feb 5, 2021). Molecular replacement was performed using PHASER from the CCP4 program suite (version 7.0). All structures were refined using PHENIX software (version 1.17). Structural models were built in Coot (version 0.8.2). All figures showing atomic models were generated using PyMol software (version 1.8.6).

For manuscripts utilizing custom algorithms or software that are central to the research but not yet described in published literature, software must be made available to editors and reviewers. We strongly encourage code deposition in a community repository (e.g. GitHub). See the Nature Portfolio guidelines for submitting code & software for further information.

Data

Policy information about availability of data

All manuscripts must include a data availability statement. This statement should provide the following information, where applicable:
- Accession codes, unique identifiers, or web links for publicly available datasets
- A description of any restrictions on data availability
- For clinical datasets or third party data, please ensure that the statement adheres to our policy

Coordinates and structure factors were deposited in the RCSB Protein Data Bank with accession codes:

- 8FC1 for the T. thermophilus 70S ribosome in complex with protein Y, hygromycin A, and erythromycin;
- 8FC2 for the T. thermophilus 70S ribosome in complex with protein Y, hygromycin A, and azithromycin;
- 8FC3 for the T. thermophilus 70S ribosome in complex with protein Y, hygromycin A, and telithromycin;
- 8FC4 for the A2058-N6-dimethylated T. thermophilus 70S ribosome in complex with protein Y, hygromycin A, and erythromycin;
- 8FC5 for the A2058-N6-dimethylated T. thermophilus 70S ribosome in complex with protein Y, hygromycin A, and azithromycin;
- 8FC6 for the A2058-N6-dimethylated T. thermophilus 70S ribosome in complex with protein Y, hygromycin A, and telithromycin.

All previously published structures that were used in this work for model building and structural comparisons were retrieved from the RCSB Protein Data Bank: PDB entries 6XHX [https://doi.org/10.2210/pdb6XHX/pdb], 5DOY [https://doi.org/10.2210/pdb5DOY/pdb], 4Z3S [https://doi.org/10.2210/pdb4Z3S/pdb], 4V7V [https://doi.org/10.2210/pdb4V7V/pdb], 7S1G [https://doi.org/10.2210/pdb7S1G/pdb], and 4Y4O [https://doi.org/10.2210/pdb4Y4O/pdb].

Human research participants

Policy information about studies involving human research participants and Sex and Gender in Research.

Reporting on sex and gender	Not Applicable
Population characteristics	Not Applicable
Recruitment	Not Applicable
Ethics oversight	Not Applicable

Note that full information on the approval of the study protocol must also be provided in the manuscript.

Field-specific reporting

Please select the one below that is the best fit for your research. If you are not sure, read the appropriate sections before making your selection.

[x] Life sciences [ ] Behavioural & social sciences [ ] Ecological, evolutionary & environmental sciences

For a reference copy of the document with all sections, see nature.com/documents/nr-reporting-summary-flat.pdf

Life sciences study design

All studies must disclose on these points even when the disclosure is negative.

Sample size	Not applicable because no experiments requiring sample size determination have been performed.
Data exclusions	No data were excluded from the analyses.
Replication	MICs were determined in biological duplicates. Time-kill assays were performed in biological triplicates. The toe-printing gel image shows one representative of two independent experiments. All attempts at replication were successful.
Randomization	MIC, checkerboard and time-kill assays were intrinsically randomized, as the experimental groups constituted equally sized aliquots of a common suspension of bacterial cells. Likewise, no extrinsic randomization was necessary for the toe-printing assay, which used a homogeneous cell-free transcription-translation mixture. For cross-validation during crystallographic model building and refinement, an R-free set was used.
Blinding	Blinding was not used in MIC and checkerboard assays because the outcome (turbidity) is not deemed subjective outside the margin of error (one binary dilution) typical for this experiment. Blinding was not used in time-kill assays because the outcome (bacterial counts) was determined by serial dilution, a quantitative technique. Blinding was not used in the toe-printing experiments because the outcome (differences in translation-arrest sites and inhibition-escape) are evident upon inspection of unprocessed gel images, and no attempt to quantify these differences was made. Blinding was not used in X-ray crystallographic experiments.

Reporting for specific materials, systems and methods

We require information from authors about some types of materials, experimental systems and methods used in many studies. Here, indicate whether each material, system or method listed is relevant to your study. If you are not sure if a list item applies to your research, read the appropriate section before selecting a response.

Materials & experimental systems

n/a	Involved in the study	
☒	☐	Antibodies
☒	☐	Eukaryotic cell lines
☒	☐	Palaeontology and archaeology
☒	☐	Animals and other organisms
☒	☐	Clinical data
☒	☐	Dual use research of concern

Methods

n/a	Involved in the study	
☒	☐	ChIP-seq
☒	☐	Flow cytometry
☒	☐	MRI-based neuroimaging
